# Development of SNP-Based Genomic Tools for the Canadian Bison Industry: Parentage Verification and Subspecies Composition

**DOI:** 10.3389/fgene.2020.585999

**Published:** 2020-11-20

**Authors:** Tianfu Yang, Michelle Miller, David Forgacs, James Derr, Paul Stothard

**Affiliations:** ^1^Department of Agricultural, Food and Nutritional Science, University of Alberta, Edmonton, AB, Canada; ^2^Neogen Canada, Edmonton, AB, Canada; ^3^Department of Veterinary Pathobiology, College of Veterinary Medicine & Biomedical Sciences, Texas A&M University, College Station, TX, United States

**Keywords:** parentage verification, subspecies composition, bison, genomic tools, SNP genotyping

## Abstract

Genomic technologies have been increasingly applied in livestock production due to their utility in production management and animal genetic improvement. The current project aimed to develop genomic resources for the Canadian bison industry, specifically a parentage verification tool and a subspecies composition tool. Both products stand to help with building and maintaining purebred and crossbred bison populations, and in turn bison conservation and production. The development of this genomic toolkit proceeded in two stages. In the single-nucleotide polymorphism (SNP) discovery and selection stage, raw sequence information from 41 bison samples was analyzed, and approximately 52.5 million candidate biallelic SNPs were discovered from 21 samples with high sequence quality. A set of 19,954 SNPs (2,928 for parentage verification and 17,026 for subspecies composition) were then selected for inclusion on an Axiom myDesign custom array. In the refinement and validation stage, 480 bison were genotyped using the custom SNP panel, and the resulting genotypes were analyzed to further filter SNPs and assess tool performance. In various tests using real and simulated genotypes, the two genomic tools showed excellent performance for their respective tasks. Final SNP sets consisting of 191 SNPs for parentage and 17,018 SNPs for subspecies composition are described. As the first SNP-based genomic toolkit designed for the Canadian bison industry, our results may provide a new opportunity in improving the competitiveness and profitability of the industry in a sustainable manner.

## Introduction

Bison meat is a growing and economically relevant industry in Canada. According to the Canadian Bison Association, the industry has seen a compounded annual growth rate of 5% since 1996, with the Canadian herd at roughly 145,000 animals as of January 1, 2017^1^. Prices have also shown a strong increasing trend since 2003, from $1.18/lb. to $5.75/lb^2^. In 2019, Canada exported more than 20,000 live bison to the United States, and its global export of bison meat was worth more than 17 million Canadian dollars^3^. A notable opportunity exists in the Canadian bison industry to apply genomic tools to assist in the operational management of bison herds. For this reason, we developed genomic tools based on single-nucleotide polymorphisms (SNPs) discovered in the American bison (*Bison bison*) for parentage verification and genome composition estimation.

Pedigree records are critical for herd management in animal production, for which parentage verification is a valuable tool. Genetic markers have been used in verifying the parentage of animals for decades. The early efforts can be traced back to the 1980s, and a variety of marker types have been used (Quinn et al., 1987; Wetton et al., 1987; Scott et al., 1992; Queller et al., 1993), differing in terms of informativeness, resolution, reproducibility, and cost (Mueller and Wolfenbarger, 1999; Nadeem et al., 2018). SNP discovery efforts, coupled with the availability of high-throughput SNP genotyping arrays, have led to the use of SNPs for parentage verification in many livestock species. For example, a set of 100 core SNPs and 100 backup SNPs makes up the set used for parentage in cattle (ISAG-ICAR SNP panel)^4^. These applications of SNPs have demonstrated their many advantages (Flanagan and Jones, 2019). However, an initial investment is required to identify and validate suitable SNPs for the development of the SNP-based genomic tools.

The American bison is composed of two subspecies, plains bison (*Bison bison bison*) and wood bison (*Bison bison athabascae*), with no reproductive isolation between them (Bork et al., 1991). The ability to assess subspecies composition is of interest to the Canadian bison industry, as it would facilitate efforts to maintain subspecies genetic integrity and to explore crossbreeding. The latter could help to manage the level of hybrid vigor and breed complementarity in commercial production (Bourdon, 1997). For farmed animals with reliable pedigree records and origin information, genome composition can be calculated in a relatively straightforward manner. Alternatively, when such information is not available, which is generally the case for bison, genetic markers can be used (Frkonja et al., 2012). Previous work in bison used restriction fragment length polymorphism (RFLP) and microsatellite markers to explore the genetic relationship between different bison populations (Bork et al., 1991; Polziehn et al., 1996; Cronin et al., 2013) and provided insights into the genetic difference between the two subspecies and the subspecies composition of hybrids. However, to date, the Canadian bison industry has not made wide use of subspecies composition analysis. As is the case with parentage verification, SNPs would offer important advantages, but informative and reliable SNPs must first be identified.

In this study, we performed high-throughput sequencing and used existing sequence data to discover candidate SNPs for parentage verification and subspecies composition analysis. We then genotyped the candidate SNPs in hundreds of additional individuals and performed simulations to refine these SNP lists and to develop a breed composition equation and score. Based on the performance of these tools on a variety of known and simulated samples, they can inform management decisions aimed at improving traits and maintaining subspecies integrity and hybrid vigor. By providing detailed information on the SNP contents of each tool and the breed composition prediction approach, we hope that the tools can be used by others and further refined through, for example, the characterization of additional reference samples.

## Materials and Methods

The development of the genomic tools in the current project proceeded in two stages: (1) SNP discovery; and (2) SNP validation and refinement. In the first stage, bison whole-genome DNA sequencing data was generated or collected, SNPs were identified, and a custom medium-density SNP panel was constructed. In the second stage, a validation bison population was genotyped using the custom SNP panel, the performance of the panel SNPs for parentage and subspecies composition estimation was evaluated, and a finalized set of SNPs was proposed. The packages used in the data analysis and related parameters can be found in Supplementary Table S1.

### Stage 1: SNP Discovery

#### Sequenced Animals

Aiming to obtain genomic information from the North American bison population, we sequenced 27 bison samples collected from Canada and the United States. Genomic DNA extraction from Bison bone and hair samples was carried out using the Qiagen BioSprint 96 DNA DNeasy extraction protocol (Qiagen, Mississauga, ON, Canada). Extracted DNA was quantified using the Qubit dsDNA HS Assay (Life Technologies, Burlington, ON, United States). Sequencing libraries were constructed according to the NEXTflex DNA Sequencing Kit protocol (Bio-O Scientific, Austin, TX, United States). Between 150 ng to 1 ug of input Bison DNA was sheared using the Covaris S2 focused sonicator (Covaris Inc., Woburn, MT, United States), achieving an average fragment size of 300 to 400 bp. Size selection of end-repaired product during library preparation followed the gel-free size selection clean up process using Agencourt AMPure XP magnetic beads (Beckman Coulter, Mississauga, ON, Canada). To enable sequencing multiplexing, adapter indices from the NEXTflex DNA Barcode kit (Bio-O Scientific) were added to the libraries with 6–10 rounds of PCR amplification. QC was performed on each library using the 2100 Bioanalyzer DNA 1000 chip (Agilent Technologies, Santa Clara, CA, United States) and Qubit dsDNA HS Assay (Life Technologies) to determine the quality and quantity of each library, respectively. 26 of the 27 libraries were sequenced using the 2 × 150 cycles paired-end sequencing workflow on the HiSeqX Ten (Illumina, San Diego, CA, United States) at the McGill University and Génome Québec Innovation Centre. One library was sequenced under the CanSeq150 project using the same workflow at the Sequencing Facility of The Center for Applied Genomics (TCAG) in the Hospital for the Sick Kids. Existing whole-genome DNA sequencing data from a further 14 bison (Forgacs et al., 2016) was included in the analysis. The resulting data set includes plains (*n* = 26) and wood (*n* = 13) bison (Table 1). The sequence reads have been deposited in the Sequence Read Archive (SRA), under BioProject PRJNA658430.

**TABLE 1:** List of sequenced bison.

ID	Type	Location	Source	# Raw reads	% Reads mapped*	Average sequencing depth
P1	Plains	Caprock Canyons State Park, TX	Existing	33,573,898	38%	0.43
P2	Plains	Caprock Canyons State Park, TX	Existing	35,711,512	44%	0.53
P3	Plains	Caprock Canyons State Park, TX	Existing	63,428,284	46%	1.01
P4	Plains	Caprock Canyons State Park, TX	Existing	71,988,836	50%	1.24
P5	Plains	the Greater Yellowstone Area	Existing	345,862,044	12%	1.21
P6	Plains	the Greater Yellowstone Area	Existing	300,481,696	26%	2.68
P7	Plains	Yellowstone National Park	Existing	171,167,934	88%	5.51
P8	Plains	Yellowstone National Park	Existing	193,911,748	87%	6.19
P9	Plains	Yellowstone National Park	Existing	285,445,650	83%	8.67
P10	Plains	Yellowstone National Park	Existing	329,693,516	84%	10.12
P11	Plains	Caprock Canyons State Park, TX	New	764,693,316	66%	23.47
P12	Plains	Caprock Canyons State Park, TX	New	843,431,132	62%	23.94
P13	Plains	Cypress Hills, SK	New	939,516,516	7%	0.48
P14	Plains	Drumheller, AB	New	1,080,363,884	8%	0.59
P15	Plains	Elk Island National Park, AB	New	872,769,454	63%	25.40
P16	Plains	Elk Island National Park, AB	New	874,658,572	67%	27.36
P17	Plains	Junction of Bow and Belly Rivers, AB	New	1,075,618,578	8%	1.68
P18	Plains	Prince Albert, SK	New	962,343,124	42%	19.78
P19	Plains	Red Rock/YNP Turner Ranch	New	899,967,488	78%	37.39
P20	Plains	Santa Catalina Island, CA	New	899,471,994	58%	25.17
P21	Plains	Santa Catalina Island, CA	New	1,268,225,668	44%	26.43
P22	Plains	Swift Current, SK	New	946,903,882	11%	0.71
P23	Plains	Unknown	New	610,183,530	11%	0.48
P24	Plains	Wind Cave National Park, SD	New	776,956,818	65%	23.71
P25	Plains	Wind Cave National Park, SD	New	853,107,274	66%	26.35
P26	Plains	Yellowstone National Park	New	973,793,586	73%	36.37
U1	Unknown	Unknown	New	540,095,712	2%	0.08
U2	Unknown	Unknown	New	838,346,982	1%	0.12
W1	Wood	Elk Island National Park, AB	Existing	12,968,260	41%	0.18
W2	Wood	Elk Island National Park, AB	Existing	17,859,638	48%	0.29
W3	Wood	Elk Island National Park, AB	Existing	58,711,530	45%	0.91
W4	Wood	Elk Island National Park, AB	Existing	75,729,836	38%	0.99
W5	Wood	Alberta, Canada	New	465,548,626	18%	2.95
W6	Wood	Athabasca Lake, SK	New	974,026,536	11%	1.23
W7	Wood	Elk Island National Park, AB	New	979,762,842	69%	34.14
W8	Wood	Elk Island National Park, AB	New	991,502,862	73%	36.54
W9	Wood	Elk Island National Park, AB	New	986,508,748	75%	37.33
W10	Wood	Unknown	New	1,013,582,276	4%	0.44
W11	Wood	Unknown	New	1,026,497,582	70%	38.42
W12	Wood	Wood Buffalo National Park	New	1,000,759,832	24%	8.75
W13	Wood	Wood Buffalo National Park	New	1,014,848,338	39%	15.27

Bison samples with an average sequence depth >5 were used for SNPs calling. *The percentage was calculated as #(Reads aligned without any bit set in 1804 in the SAM FLAG)/#(Raw Reads) ^∗^ 100%, i.e., a read was not considered as mapped if any of the following was true: (1) it was not mapped; (2) its mate was not mapped; (3) it was not the primary alignment; (4) it failed platform/vendor quality checks; or (5) it is PCR or optical duplicate.

#### Sequence Alignment and SNP Calling

**FIGURE 1:**
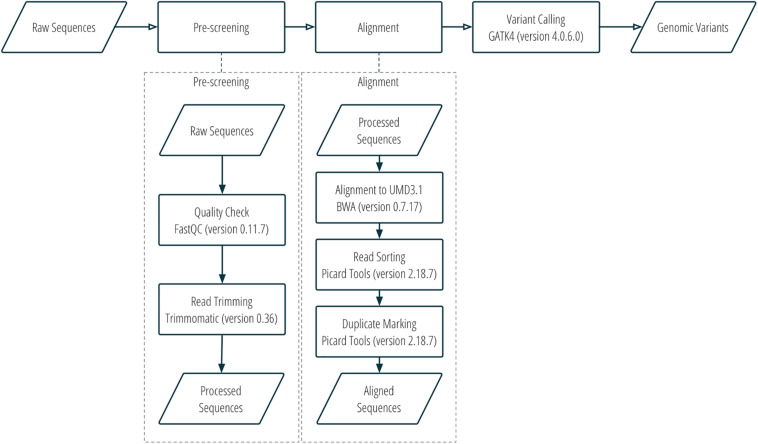
Sequence alignment and SNP calling workflow.

DNA sequence reads were assessed for quality using FastQC v0.11.7 (Andrews, 2010), trimmed with Trimmomatic v0.36 (Bolger et al., 2014), and then aligned to the bovine UMD3.1 reference genome with Burrows-Wheeler Aligner v0.7.17 (Li and Durbin, 2009). Aligned sequences were converted to bam files with Samtools v1.8 (Li et al., 2009). The bam files were then sorted, and optical duplicates were marked using Picard tools v2.18.7 (Picard Toolkit, 2019). SNP and indel variants were called using GATK4 v4.0.6.0 (Poplin et al., 2017). More details about the workflow of sequence alignment and variant calling can be found in Figure 1 and Supplementary Table S1.

#### SNP Selection and Custom SNP Panel Creation

**FIGURE 2:**
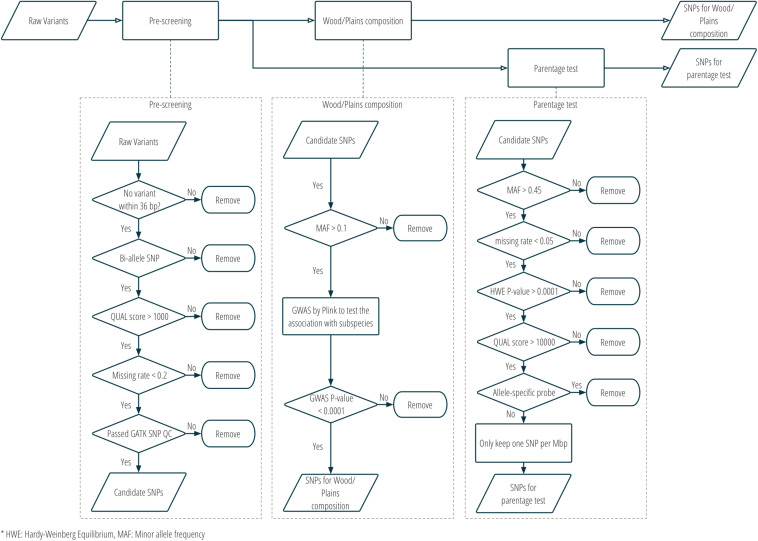
SNP selection procedure for the custom panel for parentage and subspecies composition analysis.

Two sets of SNPs were prepared for inclusion on a single custom SNP panel, one set for parentage verification, and one set for subspecies composition. More details about the SNP selection are provided in Figure 2 and Supplementary Table S1. Only bison samples with an average sequencing depth of at least 5 over the whole genome were included in the analysis for SNP selection (*n* = 21; 15 plains bison and 6 wood bison). SNPs with any of the following characteristics were removed from consideration: (1) another polymorphism exists in the 36 bp flanking sequences; (2) more than two alleles observed; (3) GATK QUAL score <1000; (4) missing rate >20%; or (5) does not pass the QC criteria recommended by GATK4^5^. The remaining SNPs were further selected based on the intended application. For parentage verification, the selection was mainly based on genotyping quality and SNP informativeness. More specifically, SNPs that met the following criteria were selected for parentage verification: (1) minor allele frequency (MAF) >0.45; (2) missing rate <5%; (3) exhibits Hardy-Weinberg Equilibrium (*p* > 0.0001); (4) QUAL score >10000; (5) requires only one probe per strand^6^. SNP thinning was conducted so that no two SNPs were located within 1 Mbp to each other, and SNPs removed during the thinning remained and served as “alternative SNPs.” For subspecies composition, SNPs were selected if the MAF was greater than 0.1 and they showed difference in allelic frequencies (nominal *p*-value < 0.0001) between the two subspecies in a genome-wide association study (GWAS). The filtering was conducted with VCFtools v0.1.15 (Danecek et al., 2011), and the GWAS was conducted with Plink v1.9 (Chang et al., 2015). Selected SNPs were submitted to Affymetrix. Those SNPs that were recommended by Affymetrix’s quality check were included in a custom Axiom SNP panel. In addition, aiming at 3,000 SNPs for parentage verification, the “alternative SNPs” were submitted for assessment, and the Affymetrix-recommended ones were added to the panel. A complete list of the parentage and subspecies-identification SNPs on the panel is provided in Supplementary Table S2.

### Stage 2: SNP Refinement and Validation

#### Animals Genotyped Using the Custom Panel

A 480 bison (including three bison sequenced during the first stage) were genotyped with the custom bison SNP panel in order to evaluate its performance. These animals came from 19 data sources from Canada and the United States. Animals with a call rate lower than 95% (*n* = 19) were excluded from subsequent analysis due to possible low sample quality. For evaluating parentage verification, there were 21 known mother-calf pairs in the dataset. For evaluating subspecies composition estimation, subspecies labels (“plains bison” or “wood bison”) were available for 291 bison with subspecies assignment confidence levels of 1 (Absolutely Confident) or 2 (Somewhat Confident). These confidence levels were solicited from the providers of the samples and ranged from 1 (Absolutely Confident) to 3 (Less Confident) (Table 2).

**TABLE 2:** Confidence level of the subspecies label in the validation population.

Confidence level	Description	Count in the validation population		
		Plains	Wood	Hybrid
1	Absolutely confident. The animal has documentation to show the origins	203	57	0
2	Somewhat confident. The animal came from a highly reliable source, but it has no documentation showing the origins	31	0	1
3	Less confident. Cannot reliably track the origins of the animal or no documentation exists regarding the origins	31	0	72
4	No data about the confidence	61	5	0

### SNPs for Parentage Verification

**FIGURE 3:**
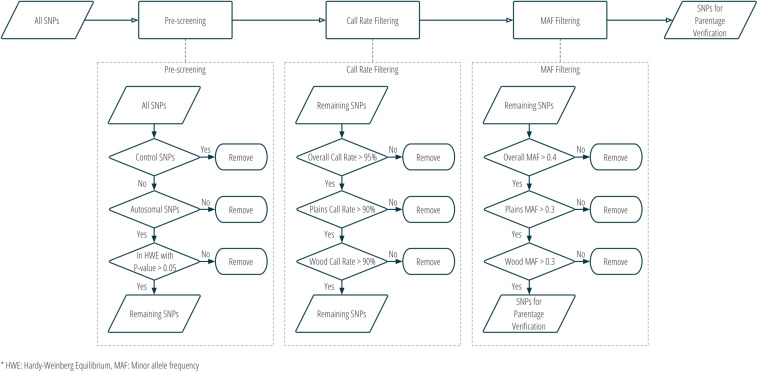
Workflow of SNP selection for parentage verification, based on genotype data from the validation population.

Although SNPs had originally been selected for two different purposes in stage one, all panel SNPs were evaluated for utility in parentage verification. Those panel SNPs that met all the following criteria were selected for parentage: (1) overall call rate >95%; (2) call rate in each subspecies >90%; (3) overall MAF >0.4; (4) MAF in each subspecies >0.3; (5) conversion type is not any one of NoMinorHom (no minor homozygote), OTV (off-target variant), or MonoHighResolution (not polymorphic)^7^; and (6) in Hardy-Weinberg Equilibrium with a nominal *p*-value > 0.05. Figure 3 and Supplementary Table S1 show the criteria used in the selection of SNPs for parentage verification in more detail.

#### Removing SNPs in linkage disequilibrium

Those SNPs that passed the screening for parentage verification were pruned to remove SNPs in linkage disequilibrium (LD). With any others since independent SNPs provide more power in parentage exclusion. The pruning was conducted using Plink v1.9 by removing the less informative SNP (i.e., with a lower MAF) in LD. The SNPs that remained in the dataset following LD pruning (*n* = 191) were treated as the final set of SNPs for parentage verification. The criteria used in the pruning can be found in Appendix 1.

#### Efficiency of parentage exclusion

For the final set of SNPs for parentage verification, a multi-locus probability of exclusion (PE) was calculated as a measurement of performance in parentage verification. Multi-locus PE is the probability to exclude (1) a random unrelated parent when the other parent is known (Q1); (2) a random unrelated parent when the other parent is unknown (Q2); or (3) a random unrelated offspring (Q3) (Dodds et al., 1996). The single-SNP PE (including Q1, Q2, and Q3) for each SNP can be calculated based on its MAF in the validation population. In order to assess the performance of our final set of SNPs for parentage verification, we explored the relationship between the number of “top SNPs” and the multi-locus PE, where “top SNPs” were defined as the SNPs with the greatest MAF in the validation population.

By applying dense SNPs in parentage verification, the multi-locus PE can be extremely close to one. In the following description and discussion, the probability of non-exclusion (PN) was used to present the efficiency of parentage tools. The relationship between PE and PN is:

PN=1-PE

PE was calculated using R^8^ and the formulae described by Dodds et al. (1996).

#### Testing with known mother-calf pairs

The final set of SNPs for parentage verification was tested with the 21 known mother-calf pairs in the validation population. The genotypes of each pair were compared to detect possible false exclusion. In addition, for each one of the 21 calves, comparisons were conducted to exclude “presumably unrelated candidates” as its father, where the “presumably unrelated candidates” were those genotyped animals coming from a different source based on available information and unlikely to be the father. The test served to evaluate the robustness of our SNP set (Tortereau et al., 2017). The comparison simulated a common parentage scenario, where the genotypes are known for both the mother and the calf, and the paternity of a putative father is to be determined (scenario Q1) (Jamieson and Taylor, 1997).

#### SNPs for Subspecies Composition

All SNPs selected at stage one went into a screening for subspecies composition SNPs, which was mainly based on genotyping quality. A SNP was deemed to be low-quality if (1) its call rate was lower than 95% in the whole validation population; (2) its call rate was lower than 90% in either subspecies; or (3) it was categorized into one of the three following conversion types during genotyping: NoMinorHom (no minor homozygote), OTV (off-target variant), or MonoHighResolution (not polymorphic)^9^.

The remaining SNPs were tested in three ways: (1) exploratory analysis: visualizing the population structure of the validation population by multi-dimensional scaling (MDS); (2) qualitative analysis: classifying bison into groups through K-means and comparing the result to their origin label, and (3) quantitative analysis: estimating the subspecies composition.

##### Multi-dimensional scaling

The utility of the selected SNPs in subspecies composition was first tested with MDS. This technique provides a way to visualize the SNP-based genetic distance between samples in a lower-dimensional space (Li and Yu, 2008). The distance was calculated as the Euclidean distance between samples based on their genotype (allele counts) of the selected SNPs. The analysis was conducted with the cmdscale function from the R stats package^10^. More details can be found in Supplementary Table S1. In the output of MDS, it was expected that bison from each subspecies would cluster together to form two distinct groups.

##### K-means clustering

Hartigan’s k-means clustering was used to test whether the selected SNPs could classify samples into two groups, corresponding to plains and wood bison, which would be strong evidence to support that the selected SNPs are informative for estimating subspecies composition. The algorithm aims to partition the samples into a specified number of clusters (two in this case) so as to minimize the within-cluster variances (squared Euclidean distances) (Saatchi et al., 2011). The analysis was performed using the k-means function from the R stats package^11^. Additional details can be found in Supplementary Table S1. The clustering result was expected to agree with the origin label of the bison.

##### Genome composition of bison in the validation population

In order to provide a quantitative measurement of the genome composition (i.e., genome proportions from plains bison and wood bison), we further developed an estimation equation based on constrained genomic regression (Boerner and Wittenburg, 2018). The plains bison with a subspecies assignment confidence level of 1 (*n* = 203) and the wood bison with a subspecies assignment confidence level of 1 (*n* = 57) were treated as reference populations, and their population allele frequencies were calculated for the selected SNPs. The estimation equation was applied to bison with a confidence level of 1 or 2 labeled as “plains bison” (*n* = 234) or “wood bison” (*n* = 57).

The *i*th bison’s genome composition was estimated with a constrained regression:

$$f_{i}=f_{P}b_{Pi}+f_{W}\left(1-b_{Pi}\right)+e,0\leq b_{P}\leq 1$$

where *f*_*i*_ = (*f*_*i*1_, *f*_*i*2_, …, *f*_*im*_) is the allele frequencies of the *m* SNPs for that bison, *f*_*P*_ = (*f*_*P*1_, *f*_*P*2_, …, *f*_*Pm*_) is the allele frequencies of the *m* SNPs in the plains reference population, *f*_*W*_ = (*f*_*W*1_, *f*_*W*2_, …, *f*_*Wm*_) is the allele frequencies of the *m* SNPs in the wood reference population, *b*_Pi_ is the genome proportion from plains bison for the *i*th bison (PlainsScore), and *e* is the residual error. Since we focused on estimating the genome composition contributed by plains and wood bison without considering other possible contributors, 1 − *b*_Pi_ is the genome proportion from wood bison for the *i*th bison. The constraint of 0 ≤ *b*_P_ ≤ 1 ensures that the genome compositions are between 0 and 1. The calculation was conducted with R/limsolve (see “text footnote 8”)^12^.

The constrained genome regression approach has three features that led us to apply it here: (1) it runs in a “supervised” mode, where the reference populations are known; (2) it does not explicitly require the SNPs to be in linkage equilibrium; and (3) it has achieved high estimation accuracy in simulation analysis (Boerner and Wittenburg, 2018).

##### Genome composition of simulated populations

In addition to the real data from the validation population, the genome composition estimation method was applied to six populations simulated using the package hybriddetective (Wringe et al., 2017). The simulated populations were: (1) pure plains; (2) pure wood; (3) F1 (plains × wood); (4) F2 (F1 × F1); (5) backcross to plains (F1 × plains); and (6) backcross to wood (F1 × wood). The genotypes in each population were simulated based on the allele frequencies in the corresponding parental populations. More details can be found in Supplementary Table S1. Each simulation population consisted of 500 animals.

## Results

### Stage 1: SNP Discovery

Twenty-seven American bison samples were sequenced for SNP discovery, and data from an additional 14 bison samples were obtained from previous studies. Raw sequence reads were mapped to the bovine UMD3.1 reference genome, and 21 samples with an average sequencing depth of at least 5 over the whole genome were used for SNP calling. The number of raw reads, read mapping percentages, and average sequence depth is given for each sample in Table 1. Approximately 62 million genomic variants were discovered from the analysis, among which around 52.5 million variants were biallelic SNPs^13^. After the first stage of SNP selection (Figure 2), 2,928 SNPs were selected as candidates for parentage verification, and 17,026 SNPs as candidates for subspecies composition. These SNPs were included on a custom Affymetrix panel, which was then used to genotype 480 bison in what we refer to as the validation population.

### SNPs for Parentage Verification

**FIGURE 4:**
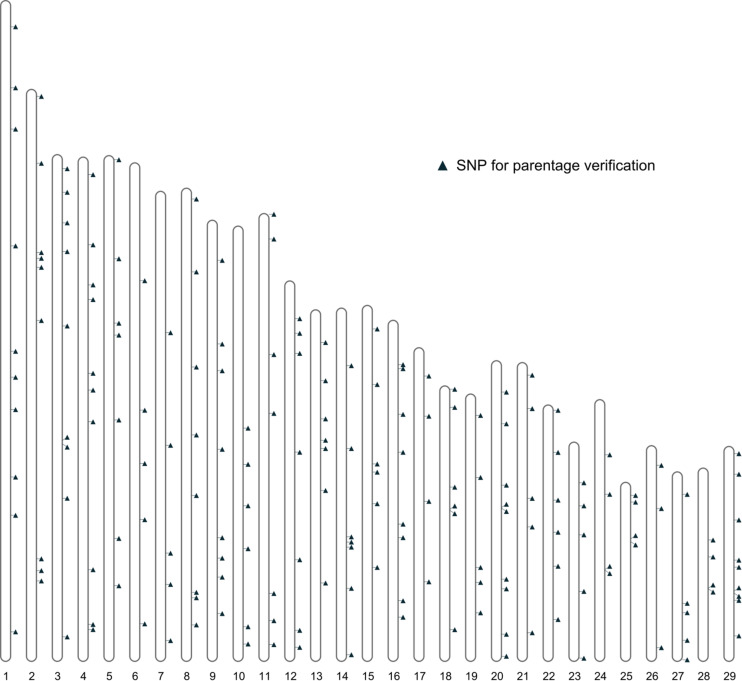
The distribution of the SNPs for parentage verification on autosomes.

Further filtering of SNPs based on MAF, genotype quality, and LD was performed using the 461 bison genotypes that passed quality checks. This filtering produced a final set of 191 SNPs deemed suitable for parentage verification. The distribution of these SNPs across autosomes is shown in Figure 4.

#### Efficiency of Parentage Exclusion

**FIGURE 5:**
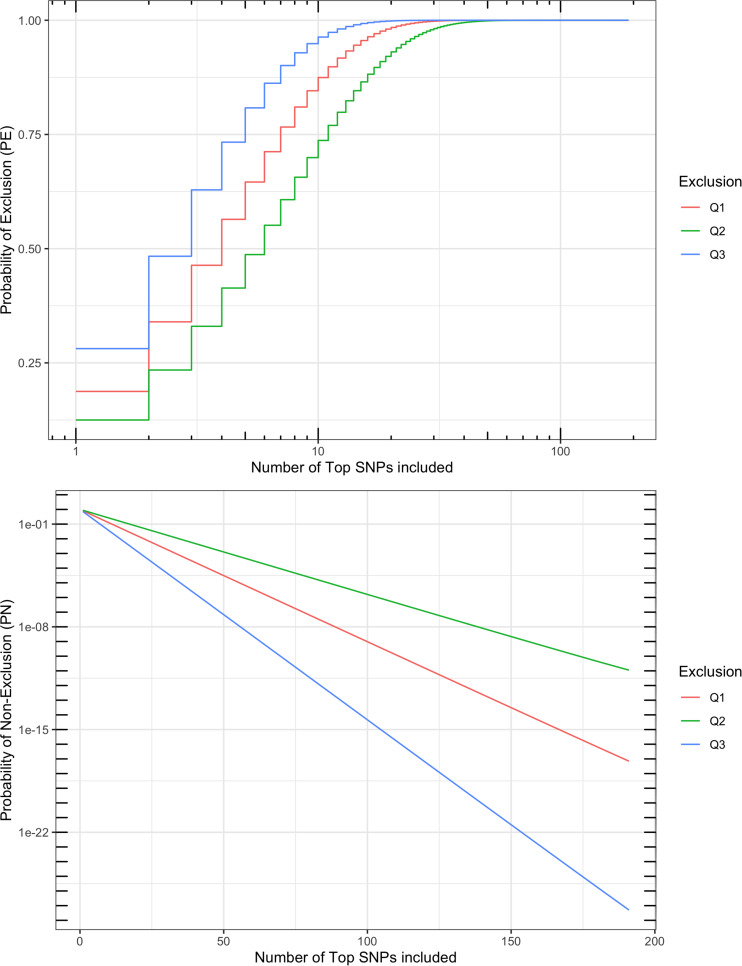
Probability of exclusion (PE) and probability of non-exclusion (PN) for different numbers of top parentage SNPs. Multi-locus PE is the probability to exclude (1) a random unrelated parent when the other parent is known (Q1), (2) a random unrelated parent when the other parent is unknown (Q2), or (3) a random unrelated offspring (Q3).

A multi-locus probability of non-exclusion (PN) was calculated for the 191 SNPs based on their MAF, as a measurement of performance in parentage verification. Three types of PNs were calculated: (1) the PN for a random unrelated parent when the other parent is known (termed “Q1”); (2) the PN for a random unrelated parent when the other parent is unknown (termed “Q2”); and (3) the PN for a random unrelated offspring (termed “Q3”). The three PNs for this SNP set were 7.0 × 10^–18^, 1.1 × 10^–11^, and 5.0 × 10^–28^, respectively (Figure 5). For comparison, the ISAG-ICAR SNP panel, a commonly used bovine parentage verification tool, achieves a PN of 7.2 × 10^–26^ for Q3, and 1.4 × 10^–10^ for Q2 on the German Holstein population (Schütz and Brenig, 2015). The comparable results suggested that the SNPs selected in our analysis are informative and suitable for parentage verification. For American bison, a microsatellite panel including 15 markers has reported a PN of 0.0266 for Q2 and 0.0024 for Q1. More recent microsatellite panels have been used in parentage testing for American bison, usually including more markers, but no PE or PN report was found.

#### Testing With Known Mother-Calf Pairs

The genotyped set of animals included 21 known mother-calf pairs. All 21 pairs were recovered with perfect concordance using the 191 SNPs. In the meanwhile, consistent with the very low PN values calculated based on MAF, the test successfully excluded all “presumably unrelated candidates” for paternity in the typical parentage testing scenario.

### SNPs for Subspecies Composition

A set of 17,018 SNPs remained for use in subspecies composition analysis after filtering based primarily on genotyping quality in the validation population. Their performance in subspecies composition estimation was evaluated with clustering techniques (MDS and k-means) and constrained genomic regression.

#### Multi-Dimensional Scaling

**FIGURE 6:**
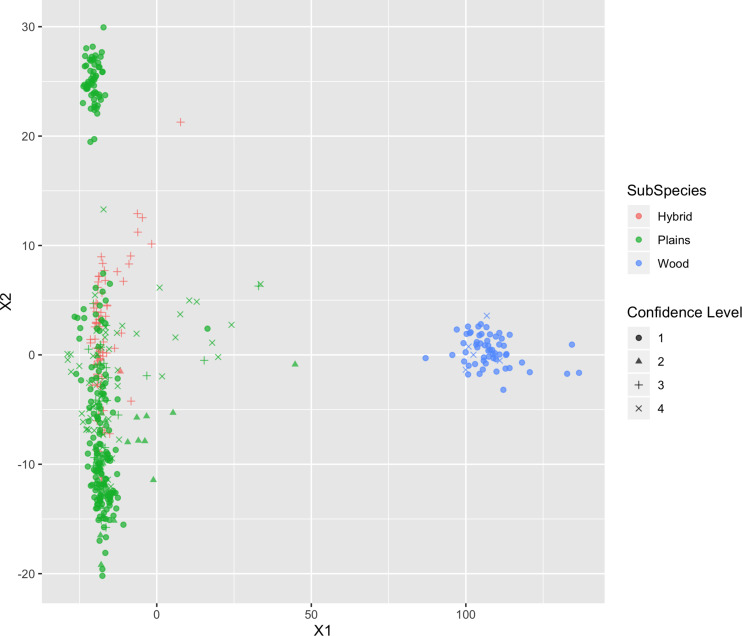
Multi-dimensional scaling (MDS) visualization of genetic distances between plains and wood bison determined using the custom panel.

**FIGURE 7:**
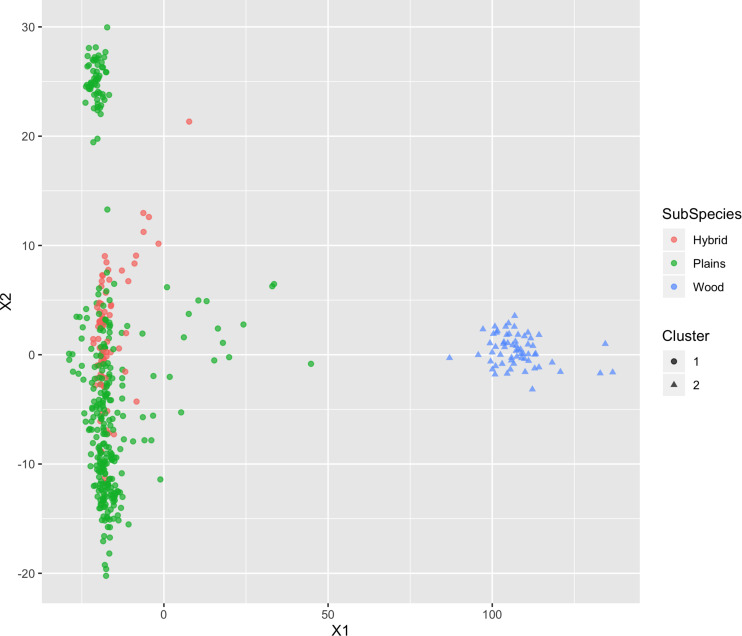
Multi-dimensional scaling (MDS) visualization of genetic distances between plains and wood bison determined using the custom panel. Cluster inference was performed by k-means clustering.

The results of MDS (Figures 6, 7) shows that the plains bison and the wood bison in the validation population visually group into three clusters in 2-dimensional space. The two clusters on the left side correspond to the plains bison samples, and the cluster on the right side corresponds to the wood bison samples. On the X1 axis, those plains bison with an assignment confidence level of 1 (absolutely confident) tend to be further away from the group of wood bison. These observations support that the two subspecies are separable using our selected SNP set. The plot also implies that the plains bison in the validation population can be further divided into two sub-populations. However, the focus of the current analysis is on subspecies composition.

Those bison labeled as hybrid did not appear to be a group between plains and wood bison. Instead, they are largely overlapping with one of the plains bison group. Given that the “Hybrid” label was almost exclusively associated with a low confidence level, these animals were not able considered to be informative when judging the effectiveness of our SNP set. Instead, further validation related to hybrid bison was conducted using a variety of simulated datasets.

#### K-Means Clustering

The k-means clustering using genotypes was also able to separate the plains bison and the wood bison. When the cluster number was set as 2, the k-means clustering assigned those bison with reliable origin (confidence level 1 and 2, *n* = 292) into two groups: One group included exactly the 57 wood bison, and the other one included 234 plains bison and 1 hybrid bison. The results suggest that the selected SNP set provides sufficient information for plains bison and wood bison composition estimation.

#### Genome Composition Estimation

**FIGURE 8:**
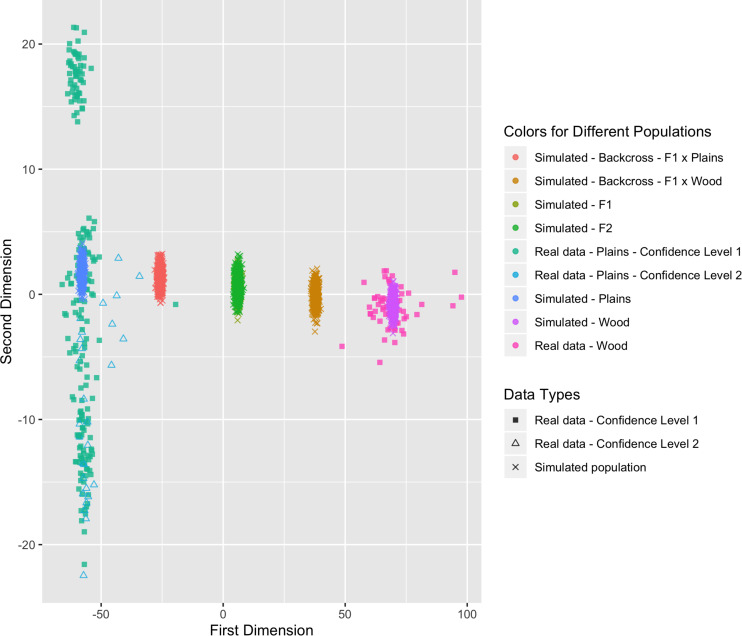
Multi-dimensional scaling (MDS) visualization of genetic distances between simulated populations determined using the custom panel.

Six populations (pure plains, pure wood, F1, F2, backcross to plains, and backcross to wood) were simulated based on the reference populations (i.e., the bison with a subspecies assignment confidence level of 1), with 500 bison simulated in each population. The genetic distance between the simulated populations and the real validation populations can be found in Figure 8. The simulated pure bison clustered around the center of the corresponding pure reference populations. The simulated F1 and F2 populations largely overlapped, and they were located in the middle between the simulated pure plains and pure wood population. The five simulated populations aligned into a line on the figure, which was expected based on their relationship. The simulation is based on population-level allele frequencies in the reference populations. As a result, the simulated pure bison populations, especially the plains bison, are more genetically homogeneous than the corresponding real populations.

These simulated animals served as a way to test the performance of the genomic composition estimation method, especially in hybrid populations. Table 3 shows the estimated genome composition from plains bison (PlainsScore) for the validation population and simulated populations. The reference plains population has a median PlainsScore of 100%, and the reference wood population has a median PlainsScore of 0.87%. These values were expected for the most reliably labeled plains and wood bison. Those bison labeled as plains with a subspecies assignment confidence level of 2 had a median PlainsScore of 98.67%. The mean of PlainsScore was more sensitive to the existence of outliers and tended to deviate more from the expected value for the three populations. For the six simulated population, their median PlainsScore and mean PlainsScore were very close to their expected values.

**TABLE 3:** Estimated subspecies composition for reference population and simulated population.

#	Population	*n*	Plains score (%)		
			Median	Mean	SD
1	Reference – Plains (Confidence level 1)	203	100.00	99.09	2.28
2	Reference – Wood (Confidence level 1)	57	0.87	2.16	3.02
3	Plains bison (Confidence level 2)	31	98.67	94.81	9.81
4	Simulated–Plains	500	99.94	99.85	0.20
5	Simulated–Wood	500	0.00	0.17	0.26
6	Simulated – F1	500	49.95	49.97	0.43
7	Simulated – F2	500	50.04	50.05	0.54
8	Simulated–Backcross–Plains	500	75.00	74.99	0.48
9	Simulated–Backcross–Wood	500	25.03	25.03	0.49

*There is no wood bison with a confidence level 2.*

## Discussion

Genomic technologies have attracted keen interest in animal producers by their potential in production management and animal genetic improvement. They have been, therefore, increasingly applied in livestock production, especially in dairy, beef, and pork industries (van der Steen et al., 2005; Van Eenennaam et al., 2014; Berry et al., 2016). Our project aims to provide two valuable genomic tools for animal management in the Canadian bison industry: parentage verification and subspecies composition estimation. Parentage verification plays an essential role in breeding management as a powerful tool for maintaining pedigree information. Reliable pedigree information will clarify the outcome of breeding and support informed decision-making, such as introducing bulls with preferred phenotypes or great genetic merit. However, for the bison industry, maintaining reliable pedigree records may be relatively challenging, partially due to the lack of artificial insemination (Dorn, 1995) and the use of multi-sire pasture breeding in some herds. A low-cost genomic tool for parentage verification would be a valuable asset. In the last two decades, parentage verification for the American bison is mostly based on microsatellites^14^ (Schnabel et al., 2000; Halbert et al., 2004; Mooring and Penedo, 2014). By applying the SNP-based tool developed in the current study, a much higher PE can be achieved thanks to a larger number of informative genetic markers (McClure et al., 2018). Other advantages of the SNP-based genomic tools may include the better reproducibility of genotyping and improved time and cost efficiency.

In this work, sequence information from 41 individuals was used to discover more than 52.5 million candidate SNPs. It is important to note that more than 13.5 million (25.7%) of these SNPs were monomorphic in the bison samples, and thus could represent fixed differences between bison and cattle. Although not of utility in this study, such SNPs could be helpful for assessing cattle introgression. The number of discovered SNPs in other studies mapping reads from a related species to the bovine genome is variable, with differences likely arising from a variety of factors including sequence divergence, the number of animals sequenced, and the sequencing and analytical approaches used. For example, more than 23 million SNPs were discovered using one Gayal (*Bos frontalis*) and the bovine genome UMD3.1 as the reference (Mei et al., 2016), and more than 35 million SNPs were detected using 52 Nellore bulls (*Bos primigenius indicus*) with the latest bovine genome ARS_UCD1.2 as the reference (Fernandes Júnior et al., 2020). The use of the bovine reference genome in this manner has drawbacks. For example, there may be reads that do not align well due to genome differences that have accumulated, making any overlapping variants undetectable. In addition, genome differences could lead to spurious variants when reads from distinct loci align to a single region on the reference. Although filtering strategies can address some of these issues, it will be worthwhile re-aligning the data from this study to a high-quality bison reference genome once available.

Our genomic tools will also help with a concern of the Canadian bison industry and non-industry individuals, which is the genetic integrity of plains bison and wood bison. Conservation goals are to maintain genetically pure bison without introgression from other species, especially cattle (Freese et al., 2007), and to maintain pure wood bison and pure plains bison. The key to the latter is to correctly distinguish pure bison for each subspecies and hybrid bison. Based on the samples available to us, the genome composition estimation tool will provide valuable information. Conversely, for bison meat production, the genome composition estimation tool will enable more accurate and reliable crossbreeding between the two subspecies, by which producers may explore the possibility of improving animal performance by exploiting heterosis. An important consideration for bison producers will be the cost of these technologies relative to the projected benefits. Given the widespread of use of parentage tests in cattle and other livestock species that employ similar numbers of SNPs [e.g., the ISAG-ICAR cattle SNP panel (see “text footnote 4”)] and the application of breed composition tools [e.g., breed base representation in dairy cattle (Norman et al., 2016)], it seems likely that these tools can be economically viable. In 2018, a genomic toolkit including both parentage and breed composition tests for cattle was priced at about CA$45 per sample in Canada^15^. This cost can reasonably be expected to go down over time due to continued advances in technology.

The two SNP-based genomic tools showed high performance in various tests conducted using a validation population (480 bison) and a simulated dataset (genotypes of 3000 bison). Compared with a previously reported parentage tool for bison (Schnabel et al., 2000), our SNP-based parentage tool achieved a higher PE (i.e., lower PN), largely due to the increase in the number of included genetic markers. When compared with a recent SNP-based parentage tool, the commonly used ISAG-ICAR SNP-based tool for cattle, our parentage tool showed comparable performance in parentage exclusion (PE and PN). For plains/wood bison composition, the genomic tool successfully distinguished those plains bison and wood bison labeled with confidence, and correctly classified all animals in the simulated purebred and crossbred populations.

The accuracy and reliability of our genomic tools can be further improved over time by integrating more testing data and reliable reference animals. The improvement can be threefold. First, as more bison are genotyped, the information about genotyping quality (e.g., call rate or reproducibility) will help to detect SNPs that are difficult to genotype correctly, which should be removed from the tools (McClure et al., 2018). Second, genotype mismatch may be detected even for parent-offspring pairs with reliable records or strong genomic evidence. SNPs showing a significantly higher rate of mismatch should be excluded since they do not show the expected inheritance pattern. For example, a SNP that does not follow Mendelian inheritance in parent-offspring pairs or trios may be affected by copy number variation. Third, including more bison with known origin into the reference population will provide a better estimation of allele frequencies in plains bison and wood bison, which should improve the accuracy of the genome composition estimation. One challenge of this current work is the limited numbers of reference samples and the need for reliable subspecies labels. Ongoing efforts to obtain high-quality samples with clear lineage information could help to refine the genome composition scores. Nonetheless, based on the hundreds of samples included in our study, the composition analysis should have utility in the Canadian bison industry populations.

## Data Availability Statement

The original contributions presented in the study are publicly available. These datasets can be found here: https://www.ncbi.nlm.nih.gov/bioproject/PRJNA658430/; https://www.ebi.ac.uk/ena/browser/view/PRJEB40501.

## Ethics Statement

Ethical review and approval was not required for the animal study because Animal Care and Use Committee approval was not obtained for this study because analyses were performed either on historical bone samples or previously isolated DNA (for whole-genome sequencing), or on hair samples obtained under standard farm management procedures from commercial bison producers (for whole-genome sequencing and genotyping by SNP panel). Bison producers in Canada follow the “Code of Practice for the Care and Handling of Bison” developed by the National Farm Animal Care Council (http://www.nfacc.ca/). The Canadian Bison Association provided written consent approving the analysis of the samples. Written informed consent was obtained from the owners for the participation of their animals in this study.

## Author Contributions

MM and PS designed the project. MM, PS, JD, and DF organized the data collection. TY, PS, and MM conducted the data analysis and product development. TY and PS drafted the manuscript. All authors contributed to assessments of results and methods during the project.

## Conflict of Interest

MM is employed by the company Neogen Canada. The remaining authors declare that the research was conducted in the absence of any commercial or financial relationships that could be construed as a potential conflict of interest.

## Footnotes

1 https://www.canadianbison.ca/resources/resources/data-and-statistics/bison-producers

2 https://www.canadianbison.ca/resources/resources/data-and-statistics/price-trends

3 https://www.canadianbison.ca/resources/resources/industry-reports/bison-market-and-supply-update

4 https://www.isag.us/Docs/Guideline-for-cattle-SNP-use-for-parentage-2012.pdf

5 https://gatk.broadinstitute.org/hc/en-us/articles/360035890471-Hard-filtering-germline-short-variants

6 http://tools.thermofisher.com/content/sfs/brochures/axiom-mydesign-genotyping-technical-note.pdf

7 More details describing the conversion types can be found in the SNPolisher User Guide at http://tools.thermofisher.com/content/sfs/manuals/SNPolisher_User_Guide.pdf

8 https://www.r-project.org/

9 More details describing those conversion types can be found in the SNPolisher User Guide at http://tools.thermofisher.com/content/sfs/manuals/SNPolisher_User_Guide.pdf

10 https://stat.ethz.ch/R-manual/R-patched/library/stats/html/cmdscale.html

11 https://stat.ethz.ch/R-manual/R-patched/library/stats/html/kmeans.html

12 https://cran.r-project.org/web/packages/limSolve/index.html

13 Not all the discovered genomic variants were polymorphic in the sequenced bison population. Since the bison sequence reads were mapped to bovine reference genome, the discovered SNPs also included those genomic loci that were fixed in the 41 sequenced bison samples (about 25.7% of the discovered SNPs).

14 https://vgl.ucdavis.edu/services/dnatyping.php

15 https://www.canadiancattlemen.ca/features/genomic-tools-for-crossbred-cattle-in-the-works/

## Appendix 1 SNP Pruning

If the square of correlations (*r*^2^) in genotype allele counts was greater than 0.015 between any two SNPs, the less informative SNP (i.e., with a lower MAF) was pruned.

When LD is measured in terms of the Pearson’s correlation in genotype allele counts, its null distribution (i.e., no LD) can be approximately treated as a Student’s *t*-distribution with degrees of freedom *n*-2.

$$t=r\sqrt{\left(n-2\right)/\left(1-r^{2}\right)}$$

In our analysis, the sample size *n* is 461. A cutoff of *r*^2^ < 0.015 pruned a SNP if it was significantly (*p* = 0.001) in correlation with any other SNPs.

## References

[B1] Andrews S. (2010). *FastQC: A Quality Control Tool for High Throughput Sequence Data.* London: ScienceOpen.

[B2] Berry D. P. Garcia J. F. Garrick D. J. (2016). Development and implementation of genomic predictions in beef cattle. *Anim. Front.* 6 32–38. 10.2527/af.2016-0005 32704858

[B3] Boerner V. Wittenburg D. (2018). On estimation of genome composition in genetically admixed individuals using constrained genomic regression. *Front. Genet.* 9:185. 10.3389/fgene.2018.00185 29896217 PMC5986875

[B4] Bolger A. M. Lohse M. Usadel B. (2014). Trimmomatic: a flexible trimmer for Illumina sequence data. *Bioinformatics* 30 2114–2120. 10.1093/bioinformatics/btu170 24695404 PMC4103590

[B5] Bork A. M. Strobeck C. M. Yeh F. C. Hudson R. J. Salmon R. K. (1991). Genetic relationship of wood and plains bison based on restriction fragment length polymorphisms. *Can. J. Zool.* 69 43–48. 10.1139/z91-007

[B6] Bourdon R. M. (1997). *Understanding Animal Breeding.* Upper Saddle River, NJ: Prentice.

[B7] Chang C. C. Chow C. C. Tellier L. C. Vattikuti S. Purcell S. M. Lee J. J. (2015). Second-generation PLINK: rising to the challenge of larger and richer datasets. *Gigascience* 4:7 10.1186/s13742-015-0047-48 PMC4342193 25722852

[B8] Cronin M. A. Macneil M. D. Vu N. Leesburg V. Blackburn H. D. Derr J. N. (2013). Genetic variation and differentiation of bison (*Bison bison*) subspecies and cattle (*Bos taurus*) breeds and subspecies. *J. Hered.* 104 500–509. 10.1093/jhered/est030 23667052

[B9] Danecek P. Auton A. Abecasis G. Albers C. A. Banks E. DePristo M. A. (2011). The variant call format and VCFtools. *Bioinformatics* 27 2156–2158. 10.1093/bioinformatics/btr330 21653522 PMC3137218

[B10] Dodds K. G. Tate M. L. McEwan J. C. Crawford A. M. (1996). Exclusion probabilities for pedigree testing farm animals. *Theor. Appl. Genet.* 92 966–975. 10.1007/BF00224036 24166623

[B11] Dorn C. G. (1995). Application of reproductive technologies in North American Bison (*Bison bison*). *Theriogenology* 43 13–20. 10.1016/0093-691X(94)00006-G

[B12] Fernandes Júnior G. A. de Oliveira H. N. Carvalheiro R. Cardoso D. F. Fonseca L. F. S. Ventura R. V. (2020). Whole-genome sequencing provides new insights into genetic mechanisms of tropical adaptation in Nellore (*Bos primigenius* indicus). *Sci. Rep.* 10:9412 10.1038/s41598-020-66272-7 PMC7287098 32523018

[B13] Flanagan S. P. Jones A. G. (2019). The future of parentage analysis: from microsatellites to SNPs and beyond. *Mol. Ecol.* 28 544–567. 10.1111/mec.14988 30575167

[B14] Forgacs D. Wallen R. L. Dobson L. K. Derr J. N. (2016). Mitochondrial genome analysis reveals historical lineages in yellowstone bison. *PLoS One* 11:e0166081. 10.1371/journal.pone.0166081 27880780 PMC5120810

[B15] Freese C. H. Aune K. E. Boyd D. P. Derr J. N. Forrest S. C. Cormack Gates C. (2007). Second chance for the plains bison. *Biol. Conserv.* 136 175–184. 10.1016/j.biocon.2006.11.019

[B16] Frkonja A. Gredler B. Schnyder U. Curik I. Sölkner J. (2012). Prediction of breed composition in an admixed cattle population. *Anim. Genet.* 43 696–703. 10.1111/j.1365-2052.2012.02345.x 23061480

[B17] Halbert N. D. Raudsepp T. Chowdhary B. P. Derr J. N. (2004). Conservation genetic analysis of the texas state bison herd. *J. Mammal.* 85 924–931. 10.1644/ber-029

[B18] Jamieson A. Taylor S. C. S. (1997). Comparisons of three probability formulae for parentage exclusion. *Anim. Genet.* 28 397–400. 10.1111/j.1365-2052.1997.00186.x 9616104

[B19] Li H. Durbin R. (2009). Fast and accurate short read alignment with Burrows-Wheeler transform. *Bioinformatics* 25 1754–1760. 10.1093/bioinformatics/btp324 19451168 PMC2705234

[B20] Li H. Handsaker B. Wysoker A. Fennell T. Ruan J. Homer N. (2009). The Sequence Alignment/Map format and SAMtools. *Bioinformatics* 25 2078–2079. 10.1093/bioinformatics/btp352 19505943 PMC2723002

[B21] Li Q. Yu K. (2008). Improved correction for population stratification in genome-wide association studies by identifying hidden population structures. *Genet. Epidemiol.* 32 215–226. 10.1002/gepi.20296 18161052

[B22] McClure M. C. McCarthy J. Flynn P. McClure J. C. Dair E. O’Connell D. K. (2018). SNP data quality control in a national beef and dairy cattle system and highly accurate SNP based parentage verification and identification. *Front. Genet.* 9:84. 10.3389/fgene.2018.00084 29599798 PMC5862794

[B23] Mei C. Wang H. Zhu W. Wang H. Cheng G. Qu K. (2016). Whole-genome sequencing of the endangered bovine species Gayal (*Bos frontalis*) provides new insights into its genetic features. *Sci. Rep.* 6 1–8. 10.1038/srep19787 26806430 PMC4726396

[B24] Mooring M. S. Penedo M. C. T. (2014). Behavioral versus genetic measures of fitness in bison bulls (*Bison bison*). *J. Mammal.* 95 913–924. 10.1644/13-mamm-a-209

[B25] Mueller U. G. Wolfenbarger L. L. (1999). AFLP genotyping and fingerprinting. *Trends Ecol. Evol.* 14 389–394. 10.1016/S0169-5347(99)01659-6 10481200

[B26] Nadeem M. A. Nawaz M. A. Shahid M. Q. Doðan Y. Comertpay G. Yıldız M. (2018). DNA molecular markers in plant breeding: current status and recent advancements in genomic selection and genome editing. *Biotechnol. Biotechnol. Equip.* 32 261–285. 10.1080/13102818.2017.1400401

[B27] Norman H. D. VanRaden P. M. Megonigal J. H. Dürr J. W. Cooper T. A. (2016). 0324 Breed base representation in dairy animals of five breeds. *J. Anim. Sci.* 94 155–156. 10.2527/jam2016-0324 26812322

[B28] Picard Toolkit (2019). *GitHub Repository*. Broad Institute Available online at: http://broadinstitute.github.io/picard/

[B29] Polziehn R. O. Beech R. Sheraton J. Strobeck C. (1996). Genetic relationships among North American bison populations. *Can. J. Zool.* 74 738–749. 10.1139/z96-084

[B30] Poplin R. Ruano-Rubio V. DePristo M. A. Fennell T. J. Carneiro M. O. Van der Auwera G. A. (2017). Scaling accurate genetic variant discovery to tens of thousands of samples. *bioRxiv* [Preprint]. 10.1101/201178

[B31] Queller D. C. Strassmann J. E. Hughes C. R. (1993). Microsatellites and kinship. *Trends Ecol. Evol.* 8 285–288. 10.1016/0169-5347(93)90256-O 21236170

[B32] Quinn T. W. Quinn J. S. Cooke F. White B. N. (1987). DNA marker analysis detects multiple maternity and paternity in single broods of the lesser snow goose. *Nature* 326 392–394. 10.1038/326392a0

[B33] Saatchi M. McClure M. C. McKay S. D. Rolf M. M. Kim J. Decker J. E. (2011). Accuracies of genomic breeding values in American Angus beef cattle using K-means clustering for cross-validation. *Genet. Sel. Evol.* 43:40. 10.1186/1297-9686-43-40 22122853 PMC3250932

[B34] Schnabel R. D. Ward T. J. Derr J. N. (2000). Validation of 15 microsatellites for parentage testing in North American bison, *Bison bison* and domestic cattle. *Anim. Genet.* 31 360–366. 10.1046/j.1365-2052.2000.00685.x 11167522

[B35] Schütz E. Brenig B. (2015). Analytical and statistical consideration on the use of the ISAG-ICAR-SNP bovine panel for parentage control, using the Illumina BeadChip technology: example on the German Holstein population. *Genet. Sel. Evol.* 47:3. 10.1186/s12711-014-0085-1 25651826 PMC4318447

[B36] Scott M. P. Haymes K. M. Williams S. M. (1992). Parentage analysis using RAPD PCR. *Nucleic Acids Res.* 20:5493. 10.1093/nar/20.20.5493 1437577 PMC334374

[B37] Tortereau F. Moreno C. R. Tosser-Klopp G. Servin B. Raoul J. (2017). Development of a SNP panel dedicated to parentage assignment in French sheep populations. *BMC Genet.* 18:50. 10.1186/s12863-017-0518-2 28549462 PMC5446718

[B38] van der Steen H. A. M. Prall G. F. W. Plastow G. S. (2005). Application of genomics to the pork industry. *J. Anim. Sci.* 83 E1–E8. 10.2527/2005.8313_supplE1x 32704858

[B39] Van Eenennaam A. L. Weigel K. A. Young A. E. Cleveland M. A. Dekkers J. C. M. (2014). Applied animal genomics: results from the field. *Annu. Rev. Anim. Biosci.* 2 105–139. 10.1146/annurev-animal-022513-114119 25384137

[B40] Wetton J. H. Carter R. E. Parkin D. T. Walters D. (1987). Demographic study of a wild house sparrow population by DNA fingerprinting. *Nature* 327 147–149. 10.1038/327147a0 3574474

[B41] Wringe B. F. Stanley R. R. E. Jeffery N. W. Anderson E. C. Bradbury I. R. (2017). hybriddetective: a workflow and package to facilitate the detection of hybridization using genomic data in r. *Mol. Ecol. Resour.* 17 e275–e284. 10.1111/1755-0998.12704 28776912

